# Case control study to identify risk factors for acute hepatitis C virus infection in Egypt

**DOI:** 10.1186/1471-2334-12-294

**Published:** 2012-11-12

**Authors:** Amr M Kandeel, Maha Talaat, Salma A Afifi, Nasr M El-Sayed, Moustafa A Abdel Fadeel, Rana A Hajjeh, Frank J Mahoney

**Affiliations:** 1 Preventive and Endemic Disease Sector, Ministry of Health and Population, Cairo, Egypt; 2 Global Disease Detection and Response, U.S. Naval Medical Research Unit No. 3 (NAMRU-3), Naval, PSC 452 Box 5000 FPO, AE, 09835, USA; 3 Division of bacterial diseases, Centers for Disease Control and Prevention, Atlanta, GA, USA; 4 Centers for Disease Control and Prevention, Jakarta, Indonesia

**Keywords:** Acute Hepatitis C, Risk factor, Egypt

## Abstract

**Background:**

Identification of risk factors of acute hepatitis C virus (HCV) infection in Egypt is crucial to develop appropriate prevention strategies.

**Methods:**

We conducted a case–control study, June 2007-September 2008, to investigate risk factors for acute HCV infection in Egypt among 86 patients and 287 age and gender matched controls identified in two infectious disease hospitals in Cairo and Alexandria. Case-patients were defined as: any patient with symptoms of acute hepatitis; lab tested positive for HCV antibodies and negative for HBsAg, HBc IgM, HAV IgM; and 7-fold increase in the upper limit of transaminase levels. Controls were selected from patients’ visitors with negative viral hepatitis markers. Subjects were interviewed about previous exposures within six months, including community-acquired and health-care associated practices.

**Results:**

Case-patients were more likely than controls to have received injection with a reused syringe (OR=23.1, CI 4.7-153), to have been in prison (OR=21.5, CI 2.5-479.6), to have received IV fluids in a hospital (OR=13.8, CI 5.3-37.2), to have been an IV drug user (OR=12.1, CI 4.6-33.1), to have had minimal surgical procedures (OR=9.7, CI 4.2-22.4), to have received IV fluid as an outpatient (OR=8, CI 4–16.2), or to have been admitted to hospital (OR=7.9, CI 4.2-15) within the last 6 months. Multivariate analysis indicated that unsafe health facility practices are the main risk factors associated with transmission of HCV infection in Egypt.

**Conclusion:**

In Egypt, focusing acute HCV prevention measures on health-care settings would have a beneficial impact.

## Background

Chronic hepatitis C virus (HCV) infection is a major cause of chronic liver disease worldwide. The World Health Organization (WHO) estimates that 3% of the world’s populations, approximately 170 million people are infected with HCV. Approximately 60% of persons with HCV infection develop chronic or lifelong infection with HCV. Persons with chronic HCV infection are at increased risk for development of chronic liver disease, including 5-20% who may develop cirrhosis. It is estimated that approximately 5% of infected persons may die from the consequences of long term infection (liver cancer or cirrhosis) [1]. HCV is spread primarily by direct contact with human blood. Risk factors most frequently associated with HCV transmission are transfusion of unscreened blood, injection drug use, unsafe injections, and other iatrogenic health care procedures. The development of diagnostic assays to screen blood and blood products has resulted in a remarkable reduction in transfusion-associated HCV infection in many developed countries [2]. Similarly, the implementation of standard precautions to prevent occupational exposure to blood has resulted in a decrease in blood-borne pathogen transmission in the health care setting [3]. Since the implementation of these measures, epidemiologic studies indicate that injection drug use is the predominant mode of transmission in developed countries [4].

While, there are numerous studies documenting the emergence of HCV as an important public health problem in developing countries, there are few studies characterizing the epidemiology of HCV transmission in those countries. Available studies suggest that HCV transmission is complex and that transfusion of unscreened blood, unsafe injections, and invasive health-care procedures remain important causes of disease transmission. While transmission associated with injection drug use is also a well-recognized risk factor, many studies suggest that most HCV infections in the developing world are attributable to health care-related exposures [5].

The epidemiology of HCV transmission in Egypt has been the subject of intense study in the two decades since HCV diagnostic assays became available. Several studies have documented a prevalence of HCV antibodies ranging from 6 to 20% in the general population [6,7]. It is generally accepted that the high prevalence of HCV infection is in part attributed to parenteral antischistosomal treatment campaigns that were conducted in the 1970s. However, epidemiologic studies suggest that HCV continues to be transmitted at relatively high rates [8]. Monitoring the incidence of HCV infections is difficult because most infections are asymptomatic and available assays do not distinguish acute from chronic or resolved infections [9]. Prospective cohort studies monitoring HCV seroconversion among susceptible persons have identified incidence rates ranging from 3.1 to 5.2 per 100,000 [10,11]. Based on these studies, it is projected that 248,000 to 416,000 infections may occur each year in Egypt.

Numerous epidemiologic studies have reported diverse exposures associated with HCV infection in Egypt, including unsafe injections, health care procedures, and various community exposures. However, there are limitations to the interpretation of these data since many are based on HCV-infected persons identified in cross-sectional seroprevalence surveys and not among patients with newly acquired infections. Without well-designed studies of patients with acute disease, it is challenging to develop prevention strategies that are focused on the most important sources of ongoing transmission. This study is designed to identify risk factors among patients with newly acquired HCV infection, focusing on various healthcare and community-level practices. It is anticipated the results will inform health officials on key areas to formulate strategies for the prevention of HCV transmission in Egypt [12].

## Methods

Between June 2007 and September 2008, case-patients and controls were enrolled from two major infectious disease hospitals in Cairo (Abassia Fever Hospital) and Alexandria (Alexandria Fever Hospital). An eligible case-patient was defined as any patient with signs and symptoms of acute hepatitis, including jaundice and the following laboratory criteria: 1. Antibodies to HCV (anti-HCV) (identified by EIA second generation kits), 2. Elevated transaminase (AST) levels with a minimum of 7 fold the upper normal limits [13]. 3. Negative serologic results for hepatitis B surface antigen (HBsAg), IgM antibodies to hepatitis B core antigen (IgM anti-HBc) and IgM-antibody to hepatitis A virus (HAV). After obtaining informed consent, 54 patients were enrolled from Abassia and 32 from Alexandria Fever hospital. The study protocol ( NAMRU3.2004.0021) was approved by the U.S. Naval Medical Research Unit No. 3 Institutional Review Board and Egyptian Ministry of Health and Population (Assurance FWA00016183) in compliance with all applicable Federal regulations governing the protection of human subjects.

Gender and age-matched controls (± 5 years) were recruited from hospital visitors not living in the case-patient households. After obtaining informed consent, eligible controls were serologically screened for anti–HCV, HBsAg, IgM anti-HBc, and IgM anti-HAV. Controls with one or more positive of the above markers were excluded and replaced. Three-four sero-negative controls were recruited for each of the 86 case-patients to allow for exclusion of controls showing one or more positive antibodies for hepatitis viruses. Sample size estimates were based on calculations to detect an odds ratio (OR) of 3, assuming a frequency of single exposure of 25% among controls and 40% among patients, with a power of 80% and a significance level of 5%.

### Serologic testing

Serologic and AST testing was conducted at the participating hospital laboratories for case-patients; U.S. Naval Medical Research Unit#3 (NAMRU-3) screened controls. All institutions used enzyme-linked immunoassays (ELISA) with commercial kits (Abbott Diagnostic Division) including the second generation kit for anti-HCV. AST and ALT were tested using BIOLABO (Les Hautes Rives, 02160 Maizy, France) kits.

### Risk factors assessment

Trained social workers interviewed case-patients and controls using a standardized questionnaire that included demographic information, specific behaviors and activities in the 6 months prior to the case-patient hospital admission date. The questionnaire was pilot tested by trained interviewers.

Potential risk factors for HCV infection were categorized into healthcare-associated exposures and high risk behaviors in the community. Healthcare-associated exposures were categorized into outpatient services including type of clinic and procedures performed at outpatients versus hospital procedures including hospital admission and procedures performed at hospital. Community-level exposures included behaviors with the potential to transmit blood-borne pathogens such as sharing toothbrushes or sharp instruments, household contact with a person with acute or chronic hepatitis, sexual contact with multiple partners and being imprisoned over the previous six months. For each potential exposure to a risk factor, the date, duration of exposure, and location were recorded.

### Statistical analyses

Univariate and matched data analyses were done with EPIINFO version 6.04 (EPI6). Individual matching by gender and age (±5 years) was performed. For variables that might influence the occurrence of acute HCV, we calculated OR, 95% confidence interval (CI), and the p- value.

To assess the simultaneous association between several exposure variables and case–control status, we employed conditional logistic regression models using SPSS ver 17. These models were used to estimate ORs as a measure of association to identify risk factors for HCV infection with attention to the exposures commonly associated with HCV transmission. Case–control status was selected as the dependent variable, while all exposure variables and potential confounders significantly related to case–control status in univariate analyses with p value <0.05 were selected as independent variables in these models In these models the coefficients for the independent variables were exponentiated to estimate adjusted ORs, and standard errors for the coefficients were used to construct confidence intervals for the adjusted ORs. Variables were removed in a stepwise fashion using a likelihood ratio test at each step to assess their importance in the model, until finally only those factors significant at a 0.05 level remained in the model. All statistical tests were interpreted in a two-tailed fashion to estimate p-values and confidence intervals. The etiologic fraction in the population (EFp) for the exposures that remained in the module was calculated using OPENEPI calculator software [14].

## Results

Eighty-six case-patients and 287 age and gender-matched controls were enrolled in the study; 80 controls were excluded after serologic testing for IgM anti-HAV (16), IgM anti-HBcAb (6), anti-HCV (56) and 2 who were positive for both anti-HAV and anti-HBcAb. The mean age of case-patients was 39.2 ±13.6 years, with slight male predominance; approximately 50% of patients were 20–39 years of age. There was no significant difference in the age, gender and distribution of occupations among case-patients relative to controls. Case-patients were more likely to be illiterate and report past medical conditions than controls (Table 1). No significant (p=0.1) difference in the risk of having the disease was found between the subjects with history of chronic medical conditions and those with no chronic conditions, excluding subjects who had had invasive procedures in the previous 6 months.

**Table 1:** Demographic characteristics of the study population

**Demographic characteristics**	**Case patients n=86**		**Controls n=287**		**OR**	**CI 95%**	**P value**
	**Mean**	**SD**	**Mean**	**SD**			
age in years	39.2	(± 13.6)	37.6	(± 12.4)	0.97	1.6-4.8	0.3
	No.	%	No.	%			
Age groups:							
< 20	2	2	8	3	0.8	0.1-4.3	0.8
20-29	23	27	77	27	1	0.6-1.8	0.9
30-39	19	22	82	28	0.7	0.4-1.3	0.3
40-49	22	26	65	23	1.2	0.7-2.1	0.7
50-59	14	16	42	15	1.1	0.6-2.3	0.7
60-69	6	7	13	4	1.6	0.5-4.7	0.4
Male	51	59	164	57	1.1	0.7-1.8	0.7
*Marital status							
Single	19	22	81	28	0.7	0.4-1.3	0.3
Married	67	78	205	72	1.4	0.8-2.6	0.2
**Education							
Illiterate	63	81	155	56	2.3	1.3-4.1	0.001
Educated	15	19	121	44	0.3	0.3-0.6	0.0000
Occupation							
Worker	17	20	60	20	0.9	0.5-1.8	0.8
Soldier	2	2	0	0	NA	NA	NA
Student	2	2	5	2	1.3	0.2-8	0.7
Employee	6	7	42	15	0.4	0.2-1.1	0.06
Medical	0	0	13	5	NA	NA	NA
Unemployed	33	38	106	37	1.1	0.6-1.8	0.8
Others	26	30	61	21	1.6	0.9-2.9	0.08
History of chronic illnesses							
Diabetes	10	12	32	12	1.1	0.5-2.3	0.9
Schistosomiasis	6	7	12	4	1.7	0.6-5.7	0.3
Hypertension	16	19	28	10	2.1	1.1-4.3	0.02
Hepatomegaly	5	6	0	0	NA	NA	NA
Spleenomegaly	2	2	0	0	NA	NA	NA
Cirrhosis	0	0	2	1	NA	NA	NA
Gall stones	21	24	39	14	2.1	1.1-3.9	0.02

* data missing for one control.

** data missing for 8 cases and 11 controls.

### Univariate analysis

At least one risk factor was detected in 80 patients; 6 patients (7%) reported they had not been exposed to any of the potential risk factors. Several outpatient medical-related procedures were significantly associated with acquiring acute HCV infections, including receiving injections with reused syringes, receiving IV fluids, minimal surgical procedures including abscess drainage, receiving dental care and wound stitches in an outpatient setting (Table 2). Visiting private, urban outpatient clinics was also associated with risk of HCV infection, whereas attending governmental, rural outpatient clinics was not. Although visiting a family planning clinic among females was associated with the disease, yet the 95% CI included 1. After stratifying with attending an outpatient clinic, only receiving IV fluid, reused syringe use and minimal surgical procedures at an outpatient clinic remained significant.

**Table 2:** Outpatient related risk factors (within 6 months) associated with transmission of acute hepatitis C virus infection, Abbassia and Alexandria Fever Hospitals, Egypt

**Exposure**	**Cases n=86**		**Controls n=287**		**OR**	**95% Cl**	**P value**
	**No**	**%**	**No**	**%**			
Receiving injections	27	31	54	19	2	1.1-3.5	0.008
Receiving injection with a reused syringe	12	14	2	1	23.1	4.7-153	< 0.000
Receiving IV fluids	30	35	18	6	8	4-16.2	< 0.000
Minimal surgical procedures (draining abscess and suturing wounds)	24	28	11	4.3	9.7	4.2-22.4	< 0.000
Visiting outpatient clinic for all causes	73	85	183	64	2.3	1.6-6.4	< 0.000
Visiting a private outpatient clinic	45	52	92	32	2.3	1.4-4	< 0.000
Visiting a rural outpatient clinic	3	3	4	1	2.5	0.4-13.8	0.1
Visiting a governmental outpatient clinic	19	22	63	22	1	0.5-2	0.5
Visit urban outpatient clinic	70	81	176	61	2.8	1.5-5.2	< 0.000
Visiting a family planning clinic (females only)	8/35	24	13/124	11	2.4	0.8-7.1	0.04
Visiting a dental outpatient clinic	28	33	61	21	1.7	1-3.2	0.02

In-patient hospital exposures were also associated with HCV infection, including hospitalization for any reason, receipt of IV fluids, surgery, endoscopy, receipt of a transfusion, labor for females and insertion of urinary catheters (Table 3). Among patients and controls who were hospitalized in the previous six months, using t-test for comparing 2 means, there was no significant difference regarding mean number of hospital admissions (p value =0.07) and mean length of stay at hospital (p value =0.06). Stratifying with hospital admission, only receiving IV fluid at hospital remained significant.

**Table 3:** Hospital-related risk factors associated with transmission of acute hepatitis C virus infection, Abbassia and Alexandria Fever Hospitals, Egypt

**Exposure**	**Cases n=86**		**Controls n=287**		**OR**	**95% Cl**	**P value**
	**No**	**%**	**No**	**%**			
Hospital admission	36	42	24	8	7.9	4.2-15	< 0.000
Receiving IV Fluid	22	26	7	2	13.8	5.3-37.2	< 0.000
Receive invasive procedure during hospitalization	22	27	14	5	6.7	3.1-14.7	< 0.000
Surgery	16	19	9	3	7.1	2.8-18.2	< 0.000
Endoscopy	5	6	3	1	5.8	2.1-31.6	0.01
Labor (for females)	5 / 35	16	5 /124	4	4	1.1-11.6	0.03
Insertion of urinary catheter	4	5	2	1	6.9	1.1-55.6	0.02
Receiving blood transfusion	7	8	2	1	12.6	2.3-89.8	< 0.000

For community-level exposures, case-patients were more likely to report injecting drug use and recent incarceration than controls. Sharing toothbrushes, use of a public barber, injury while being shaved, and receipt of injections outside the formal health care setting were not associated with HCV infection (Table 4).

**Table 4:** Lifestyle related risk factors associated with transmission of HCV

**Exposure**	**Cases n=86**		**Controls n=287**		**OR**	**95% confidence limit**	**P value**
	**No**	**%**	**No**	**%**			
Being imprisoned	6	7	1	0.3	21.5	2.5-479.6	< 0.000
Injecting drug use	20	23	7	2	12.1	4.6-33.1	< 0.000
Sharing tooth brushes	8	9	15	5	1.9	0.9-4.9	0.09
Shaving at a barber shop (for males)	50 / 51	98	157 / 163	96	1.9	0.2-43.1	0.3
Wounded at barber	21 /51	41	70 / 163	43	0.9	0.5 – 1.8	0.4
Receiving injections outside healthcare settings (home or pharmacy)	24	28	86	30	0.9	0.5-1.6	0.4

### Multivariate analysis for risk factors

Logistic regression analysis identified six independent variables (Table 5). Factors were classified into three categories: hospital exposure, outpatient care and lifestyle-associated practices. Variables in the multivariate model remained significant after adjusting for occupation, education and history of chronic diseases.

**Table 5:** Multivariate analysis module

**Risk factors**	**OR**	**CI**^**a**^	**EFp**^**b**^	**CI**^**c**^
Hospital exposures				
Receipt of IV fluids	13.7	5.6-33.5	70.3	51.2-89.5
Hospital admission	7.8	4.3-14.3	52.4	37.5-67.3
Invasive procedures ^d^	4.7	2.8-7.9	35.1	23.7-46.5
Outpatient care				
Abscess drainage	33.4	4.2-267.9	87.3	63.7-100
Receiving injection with a Reused syringes	23.1	4.7-153	82	58.9-100
Lifestyle-associated				
IV drug user	12.1	4.9-29.8	67.9	47.5-88.5

^a^ Confidence interval for the odds Ratio.

^b^ EFp is the etiologic fraction in the population.

^c^ Confidence interval for the etiologic fraction in the population.

^d^ Invasive procedures include minimal surgical procedures at outpatients and procedures at hospital.

We estimated the adjusted etiologic fraction in the population (EFp) for the six risk factors that entered the multivariate logistic regression model (Table 5). Results show that hospital exposures and invasive procedures account for the major proportion of HCV infections in Egypt.

## Discussion

For clinical diagnosis of patients with signs or symptoms of liver disease CDC recommends testing by using anti-HCV [15]. However anti-HCV testing detects the presence of antibodies to the virus, indicating exposure to HCV and cannot distinguish between someone with an active or a previous HCV infection. Before 2004 when CDC developed a case definition for acute hepatitis, all studies searching for HCV infection risk factors were using anti-HCV testing to identify cases, this means that using this case definition might not reflect the accurate risk factors for infection because it is unclear when the patient was exposed. This is the first study to use the case definition developed by CDC in 2004 [13] to enroll the acute newly infected cases to help identify accurately the true risk factors for HCV infection.

Primary prevention of HCV infection remains the most important strategy to prevent HCV complications. This is particularly important due to absence of vaccine and cost effective medical therapy. The impact of any effort to prevent and control HCV infections relies on accurate epidemiological data, particularly the identification of risk factors associated with new infections. Egypt, like other countries with a similar disease burden, have limited funds to support wide-scale prevention programs. Therefore, targeting and prioritizing prevention activities are essential [12].

Most seroprevalence studies in Egypt suggest community-acquired practices account for the bulk of HCV transmission in Egypt [16]. This has lead to considerable investment in communication strategies directed at the public to promote behavior changes to decrease sharing razors and toothbrushes, getting tattoos and other risky behaviors. Identifying current risk factors for ongoing HCV transmission is challenging, as most HCV infections are initially asymptomatic and available assays do not distinguish acute from chronic or resolved infections. The US Centers for Disease Control and Prevention (CDC), in association with the Council of State and Territorial Epidemiologists (CSTE), recently developed a case definition for acute hepatitis C to differentiate between recent infections, old infections and disease exacerbation [13]. The publication of this case definition encouraged us to conduct a case control study to explore risk factors for HCV infection among patients meeting the case definition of acute disease. Other methods to identify patients with newly acquired HCV infection include identification of patients who seroconvert from negative to positive anti-HCV antibody. Cohort studies have identified such patients in village-based studies. However, the number of patients is small and the ability to identify risk factors that are generalizable has been limited [17].

The findings from this study highlight the importance of health care exposures as a source of ongoing HCV transmission in Egypt. The findings suggest both outpatient procedures and as well as exposures within the hospital play a role in transmission. The risks in the outpatient settings are apparent, particularly if providers reuse injection equipment e.g. syringes and IV line. Of particular concern is the apparent break in standard precautions during minor procedures such as administration of IV fluids, suturing, and conducting minimal surgeries. This suggests that primary care staff lack the basic skills for using safe procedures such as the use of aseptic techniques, or it could be due to a shortage of supplies. In this study, outpatient dental care was not found to be strongly associated with HCV infection. Population-based or larger case–control studies are needed before any firm conclusions can be inferred regarding the role of dental care and HCV transmission.

The possibility of acquiring acute HCV infections through hospitalization for any cause has been the subject of intense study [18-20], particularly in developing countries. The rapid introduction of new technologies and invasive procedures in settings without well-developed infection control programs provides a unique ecologic niche for HCV transmission [19]. This is particularly problematic in countries like Egypt where the prevalence of HCV infection among hospitalized patients is high and minor lapses in standard precautions can expose health care workers and patients to HCV-infected blood or body fluids [21].

In this study, we observed that several invasive procedures were associated with acquiring new HCV infections. Prior studies have identified surgery or invasive medical procedures as main risk factors associated with transmission of HCV infection in developed countries [20,22]. In a case–control study in Italy, Mele et al. identified an invasive procedure within 6 months of acute HCV infection onset in 25% of the patients [23].

The association of HCV infection with endoscopy combined with interventional procedures such as biopsies or scelerotherapy, has also been described [24]. In a 1994–1999 study performed by the Italian National Hepatitis Surveillance System [23], endoscopy had been performed in 4.6% of the patients with acute HCV infection and the estimated OR for endoscopy was 2.1 (95% CI, 1.2–3.6). This has implications for Egypt, due to the high number of patients with chronic hepatitis from schistosomiasis and chronic hepatitis B requiring endoscopy. Risk of transmission of HCV through endoscopy is easily eliminated when the recommended cleansing, decontamination and sterilization procedures are followed [25-27].

In addition to exposures in the health care setting, this study also identified injection drug use as an independent risk factor for HCV infection. In the developed world, there is accumulated evidence that injection drug use is the predominant source of new HCV infections over the past few decades which accounts for 68% and 80% of current infections [2,28,29]. While this study also highlights the role of injecting drug use as a risk factor for HCV infection in Egypt, the percentage of patients reporting injection drug use (23%) is considerably low in comparison to developed countries. Egypt is among the African countries that recently reported experiencing injection drug use. Little information regarding drug use is available in Egypt not only because it is relatively new, but also because of the lack of funds to monitor it in a systematic way [30]. This study also found that incarceration six months prior to start of illness was also a risk factor for disease. Further studies are needed to identify the specific source of HCV infection among incarcerated persons.

We did not identify any evidence of occupational or sexual risk factors associated with HCV transmission in Egypt. Numerous studies suggest that HCV transmission through occupational and sexual exposures is inefficient and that these exposures are unlikely to be major sources of new HCV infections, regardless of the population or geographic location [2].

Egypt is now experiencing a wave of HCV-related morbidity with increasing numbers of patients with end stage cirrhosis or hepatocellular carcinoma. The cost of treatment or liver transplantation is a strain on the national health care budget. Many patients are presenting at the peak of their productive lives with considerable impact on their families and community. Preventing ongoing transmission is of paramount importance to the health of future generations. Therefore, national strategies should focus on primary prevention by reducing the numbers of new infections. Ideally, successful treatment of infected patients has the potential to help reduce the incidence of HCV infection by decreasing the reservoir of infected persons who can serve as a source of transmission [10]. However, in Egypt the burden of disease is high and the capacity to treat large numbers of patients to reduce viral load is limited. This may reduce the possibility of having a meaningful impact on the reservoir of HCV-infected people in Egypt through treatment regimens.

Limitations of the study include selection of controls from the hospital visitors of the enrolled case patients or other patients in the same hospital; this may lead to selection bias because the cases and controls were not matched by place of residence.

## Conclusion

The results of this study show that a number of newly identified risk factors are contributing to HCV transmission in Egypt. As HCV prevention remains a major challenge, and due to limited resources, activities to stop transmission should focus on safe practices in the health-care setting. A national program to promote infection control has been implemented in Ministry of Health Hospitals since 2002, which was expanded to all general and district hospitals (356 hospitals). They have plans to expand further to primary care hospitals and units [31]. However, the healthcare system in Egypt is huge and complex and requires human and financial resources to strengthen infection control programs in settings where they exist and expand them to involve all healthcare institutions in Egypt, such as university, private, military hospitals and others. Empowering community members to refuse unsafe medical procedures could be implemented. Special attention to institutionalizing infection control programs in primary outpatient care settings is also essential.

## Competing interest

Authors hereby certify that there is no conflict of interest or appearance of conflict of interest in NAMRU-3 staff professional relationships with other organizations.

## Authors’ contribution

AK participated in data collection and writing first draft of the manuscript. MT participated in study design, development of data collection tools, participated in supervision of the field work and did a lot of writing of the manuscript. SA supervised the field work and the data collection process, analyzed the data, and conducted the literature search. NE general advisor and provided final revision and approval of the version to be published. MA suggested and conducted laboratory techniques, organized and reported data. RH supervised and organized the course of the project and the manuscript. FM Study design, reviewed data collection tools, and revision and final editing of the manuscript for intellectual content before submission.

## Pre-publication history

The pre-publication history for this paper can be accessed here:

http://www.biomedcentral.com/1471-2334/12/294/prepub
